# The Effect of the COVID-19 Pandemic on Health Care Workers’ Anxiety Levels: Protocol for a Meta-Analysis

**DOI:** 10.2196/24136

**Published:** 2020-11-18

**Authors:** Lunbo Zhang, Ming Yan, Kaito Takashima, Wenru Guo, Yuki Yamada

**Affiliations:** 1 Graduate School of Human–Environment Studies Kyushu University Fukuoka Japan; 2 Faculty of Arts and Science Kyushu University Fukuoka Japan

**Keywords:** COVID-19, health care worker, anxiety, meta-analysis, review, protocol, mental health, literature, bias

## Abstract

**Background:**

The COVID-19 pandemic has been declared a public health emergency of international concern; this has caused excessive anxiety among health care workers. In addition, publication bias and low-quality publications have become widespread, which can result in the dissemination of unreliable findings.

**Objective:**

This paper presents the protocol for a meta-analysis with the following two aims: (1) to examine the prevalence of anxiety among health care workers and determine whether it has increased due to the COVID-19 pandemic, and (2) to investigate whether there has been an increase in publication bias.

**Methods:**

All related studies that were published/released from 2015 to 2020 will be searched in electronic databases (Web of Science, PubMed, PsyArXiv, and medRxiv). The risk of bias in individual studies will be assessed using the STROBE (Strengthening the Reporting of Observational Studies in Epidemiology) checklist. The heterogeneity of the studies will be assessed using the I^2^ statistic. The effect size (prevalence rates of anxiety) and a 95% CI for each paper will also be calculated. We will use a moderator analysis to test for the effect of COVID-19 on health care workers’ anxiety levels and detect publication bias in COVID-19 studies. We will also assess publication bias using the funnel plot and Egger regression. In case of publication bias, if studies have no homogeneity, the trim-and-fill procedure will be applied to adjust for missing studies.

**Results:**

Database searches will commence in November 2020. The meta-analysis will be completed within 2 months of the start date.

**Conclusions:**

This meta-analysis aims to provide comprehensive evidence about whether COVID-19 increases the prevalence of anxiety among health care workers and whether there has been an increase in publication bias and a deterioration in the quality of publications due to the pandemic. The results of this meta-analysis can provide evidence to help health managers to make informed decisions related to anxiety prevention in health care workers.

**International Registered Report Identifier (IRRID):**

PRR1-10.2196/24136

## Introduction

### Background and Research Questions

The COVID-19 pandemic has affected more than 18.9 million individuals and resulted in over 709,000 deaths globally [1]. It has, therefore, been declared a public health emergency of international concern [2]. To tide over this crisis, it is important to maintain an adequate health care workforce, which requires not only an sufficient number of health care workers but also the maximization of each health care worker’s ability to care for a greater number of patients. Since the outbreak can last several months, it is also critical that health care workers are able to perform to their full potential over an extended time interval [3].

The COVID-19 pandemic has affected many aspects of people’s lives, especially their mental health [4-7]. While health care workers have to concurrently cope with the societal shifts and emotional stressors faced by the general population, they additionally face greater risks of exposure, extreme workloads, moral dilemmas, and rapidly evolving practice environments that differ greatly from what they are familiar with [8,9]. Moreover, facing hitherto unknown challenges in both physical and mental health causes excessive tension and anxiety in health care workers [10]. While anxiety is a common mental condition that can cause emotional distress, obsessive thinking, and compulsive behavior, long-term anxiety results in psychological distress and even affects the daily lives of individuals [11]. Anxiety also impairs the executive functions that underlie our ability to control and focus on our thoughts [12]. Consequently, studying and accurately grasping the anxiety levels of health care workers is necessary to take more appropriate and corrective measures to deal with public health and safety.

Although some researchers have investigated health care workers’ anxiety levels during the COVID-19 pandemic [13,14], many new papers on COVID-19 are being released rapidly since the pandemic still poses a serious threat. The present meta-analytic study includes the latest papers, and aims to generate a more comprehensive understanding of the prevalence of anxiety among health care workers. Furthermore, to date, a comparison has not been established between studies on health care workers’ anxiety levels, related and unrelated to COVID-19. In the current outbreak situation, will studies conducted in two different periods have different effect sizes? Will levels of anxiety increase significantly? Accordingly, the first aim of our meta-analysis is to examine health care workers’ anxiety status and determine the COVID-19 pandemic’s influence by comparing COVID-19–related studies with unrelated studies.

In addition, since the onset of the outbreak, knowledge about COVID-19 is direly needed, and medical journals have drastically accelerated the publication process for COVID-19–related articles to accelerate knowledge acquisition [15,16]. In this situation, the preference for publishing papers with significant results may be more extreme, which may seriously compromise the ability to draw valid conclusions from the published literature. Since the publication bias may be highly flawed, the second aim of our meta-analysis is to investigate publication bias by comparing unpublished preprints on COVID-19 with published journal papers about COVID-19.

### Hypotheses

We have generated the following two hypotheses:

1. COVID-19 makes health care workers more anxious and thus the studies related to COVID-19 will have a larger effect size. We will investigate this by comparing studies related to COVID-19 vs studies unrelated to it.
2. Publication bias in COVID-19–related studies is widespread. We will investigate this by comparing unpublished preprints about COVID-19 with published journal papers about the disease.

## Methods

### Search Strategy

This study will follow the PRISMA (Preferred Reporting Items for Systematic Reviews and Meta-Analyses) guidelines [17]. We will search through electronic databases—Web of Science, PubMed, PsyArXiv, and medRxiv—for all published journal papers (related vs unrelated to COVID-19) and preprints (relevant to COVID-19), whose titles and abstracts include the search terms presented in Textbox 1.

**Search terms.**

(“Health Personnel” OR “Personnel, Health” OR “Health Care Providers” OR “Health Care Provider” OR “Provider, Health Care” OR “Providers, Health Care” OR “Healthcare Providers” OR “Healthcare Provider” OR “Provider, Healthcare” OR “Providers, Healthcare” OR “Healthcare Workers” OR “Healthcare Worker” OR “Health Occupations” OR “Health Occupation” OR “Health Professions” OR “Health Profession” OR “Profession, Health” OR “Professions, Health” OR “Health professions”)

AND

(Anxiety OR Hypervigilance OR Nervousness OR “Social Anxiety” OR “Anxieties, Social” OR “Anxiety, Social” OR “Social Anxieties”)

### Inclusion and Exclusion Criteria

Studies will be included only if they meet the following inclusion criteria: (1) written in English (which will be decided based on the research team’s unified considerations); (2) related to “anxiety among health care workers”; (3) quantitative research designs; (4) submitted during 2015 to 2020; (5) include standardized measures of anxiety with published psychometric data and reasonable evidence of reliability and validity; (6) include a clear description of methods used to assess and score standardized measurement instruments; and (7) include publicly available effect sizes (prevalence) or values that can be calculated (the number of health care workers with anxiety and the sample size).

The exclusion criteria are: (1) studies with insufficient data, (2) duplicate sources, (3) research with unclear methods, and (4) publications about other outbreaks.

### Data Extraction

First, duplicate papers that are found in multiple databases will be removed. Subsequently, screening of the titles and abstracts will be conducted, and papers will be removed based on the inclusion and exclusion criteria. Furthermore, the full text of the papers will be checked, and article information will be extracted using a preprepared extraction table that includes the article’s title, authors’ names, scales used, year of submission, country, sample size, whether the study has been published, whether the study relates to COVID-19, and the effect size (prevalence of anxiety). The article review and data extraction processes will be performed independently by two of the authors. When there is a disagreement between them, the other authors will resolve the conflict.

### Study Assessment Criteria

We will use the STROBE (Strengthening the Reporting of Observational Studies in Epidemiology) checklist to assess the quality of observational studies [18]. The checklist consists of 6 scales—title, abstract, introduction, method, results, and discussion—each of which includes multiple items, comprising a total of 32 items. Each item is scored as 0 (not fulfilled) or 1 (fulfilled). In the modified STROBE, scores range from 0 to 32, with scores ≥16 indicating a low risk of bias and scores <16 indicating a high risk of bias. Papers that exhibit a low risk of bias will be selected for the analysis.

### Statistical Analysis

First, the heterogeneity of the studies will be determined using the I^2^ statistical index, which ranges from 0 to 100; the larger the index, the more heterogeneous are the findings. The categories encompassed by the index will be defined based on the test developed by Higgins et al [19] to measure the extent of heterogeneity: low (25%), moderate (50%), and high (75%). A study with a heterogeneity >50% prompts the use of random effects models. For each research, we will calculate the effect size (prevalence rates of anxiety) and a 95% CI around the effect size. For the data reported, if the original paper does not list the effect size or the number of health care workers with anxiety (which can be used to calculate the effect size), the authors of the paper will be contacted and asked to provide this information. If they are unable to do so, the study will be excluded from the analyses.

Subsequently, we will use a moderator analysis to test for the effect of COVID-19 on health care workers’ anxiety levels (related vs unrelated to COVID-19), and publication bias in COVID-19 studies (preprints vs published journal papers). We will also assess publication bias using the funnel plot and Egger regression [20]. For the Egger regression, a *P* value less than the significance level (**α**=.05) suggests that publication bias is present. If publication bias is present, and studies have no homogeneity, the trim-and-fill procedure will be applied to adjust these missing studies [21].

Finally, sensitivity analyses will be performed to assess the influence of each individual study on the pooled effect size. The statistical significance level is defined as **α**=.05.

## Results

Database searches will commence in November 2020. The meta-analysis will be completed within 2 months.

## Discussion

This paper presents a protocol for a meta-analysis that aims to provide comprehensive evidence about whether the COVID-19 pandemic increases the prevalence of anxiety among health care workers and whether there has been an increase in publication bias and a deterioration in the quality of publications due to the pandemic. The results of this meta-analysis can provide evidence to help health managers to make informed decisions for preventing anxiety in health care workers.

## Abbreviations

PRISMA: Preferred Reporting Items for Systematic Reviews and Meta-Analyses
STROBE: Strengthening the Reporting of Observational Studies in Epidemiology
